# Coronary Calcium Characteristics as Predictors of Major Adverse Cardiac Events in Symptomatic Patients: Insights From the CORE320 Multinational Study

**DOI:** 10.1161/JAHA.117.007201

**Published:** 2019-03-16

**Authors:** Mallory S. Lo‐Kioeng‐Shioe, Andrea L. Vavere, Armin Arbab‐Zadeh, Joanne D. Schuijf, Carlos E. Rochitte, Marcus Y. Chen, Matthias Rief, Klaus F. Kofoed, Melvin E. Clouse, Arthur J. Scholte, Julie M. Miller, Aisha Betoko, Michael J. Blaha, Christopher Cox, Jaap W. Deckers, Joao A. C. Lima

**Affiliations:** ^1^ Department of Cardiology Johns Hopkins Hospital and School of Medicine Baltimore MD; ^2^ Department of Cardiology Erasmus Medical Center Erasmus University Rotterdam Rotterdam the Netherlands; ^3^ Toshiba Medical Systems Europe BV Zoetermeer the Netherlands; ^4^ Department of Cardiology InCor Heart Lung and Blood Institute University of Sao Paulo Medical School Sao Paulo Brazil; ^5^ National Heart Lung and Blood Institute National Institutes of Health Bethesda MD; ^6^ Department of Radiology Charité Medical School Humboldt Berlin, Germany; ^7^ Department of Cardiology Heart Center University of Copenhagen Copenhagen Denmark; ^8^ Department of Cardiology Beth Israel Deaconess Medical Center Harvard University Boston MA; ^9^ Department of Cardiology Leiden University Medical Center Leiden the Netherlands; ^10^ Johns Hopkins Bloomberg School of Public Health Baltimore MD

**Keywords:** calcium density, cardiac computed tomography, coronary artery calcium, coronary artery disease, prognosis, Computerized Tomography (CT), Prognosis, Coronary Artery Disease

## Abstract

**Background:**

The predictive value of coronary artery calcium (CAC) has been widely studied; however, little is known about specific characteristics of CAC that are most predictive. We aimed to determine the independent associations of Agatston score, CAC volume, CAC area, CAC mass, and CAC density score with major adverse cardiac events in patients with suspected coronary artery disease.

**Methods and Results:**

A total of 379 symptomatic participants, aged 45 to 85 years, referred for invasive coronary angiography, who underwent coronary calcium scanning and computed tomography angiography as part of the CORE320 (Combined Noninvasive Coronary Angiography and Myocardial Perfusion Imaging Using 320 Detector Computed Tomography) study, were included. Agatston score, CAC volume, area, mass, and density were computed on noncontrast images. Stenosis measurements were made on contrast‐enhanced images. The primary outcome of 2‐year major adverse cardiac events (30 revascularizations [>182 days of index catheterization], 5 myocardial infarctions, 1 cardiac death, 9 hospitalizations, and 1 arrhythmia) occurred in 32 patients (8.4%). Associations were estimated using multivariable proportional means models. Median age was 62 (interquartile range, 56–68) years, 34% were women, and 56% were white. In separate models, the Agatston, volume, and density scores were all significantly associated with higher risk of major adverse cardiac events after adjustment for age, sex, race, and statin use; density was the strongest predictor in all CAC models. CAC density did not provide incremental value over Agatston score after adjustment for diameter stenosis, age, sex, and race.

**Conclusions:**

In symptomatic patients, CAC density was the strongest independent predictor of major adverse cardiac events among CAC scores, but it did not provide incremental value beyond the Agatston score after adjustment for diameter stenosis.

**Clinical Perspective**

**What Is New?**

- In our symptomatic population referred for coronary angiography, Agatston score, calcium volume score, and calcium density score are significant and independent predictors of incident 2‐year major adverse cardiac events.
- Calcium density is superior to Agatston and calcium volume scoring, but it did not provide incremental value beyond the Agatston score after adjustment for diameter stenosis.

**What Are the Clinical Implications?**

- Evaluation of specific characteristics of coronary artery calcium (CAC), such as the calcium density score, may be of significance when evaluating current CAC scoring systems and assessing the role of CAC scores in coronary heart disease risk prediction models.
- Further research into associations between CAC scores and cardiac events, as well as the incremental value of specific CAC characteristics beyond the Agatston score in coronary heart disease risk prediction, is necessary to assess how broader CAC scoring may enhance risk prediction of cardiac events.

## Introduction

In patients, risk stratification of cardiovascular disease and coronary heart disease is critical for determining clinical management. Coronary artery calcium (CAC) is a component of atherosclerosis almost solely found in atherosclerotic arteries.^1^ Measured by noncontrast cardiac‐gated computed tomography (CT), CAC has proved to be an important subclinical predictor of incident cardiovascular disease^2^ and scoring of CAC is considered one of the best subclinical cardiovascular disease measures for risk prediction of cardiovascular events.^1^, ^3^, ^4^ Together with traditional risk factors, CAC scoring can be used to calculate an accurate estimate of the 10‐year coronary heart disease risk.^5^ Conversely, absence of CAC is associated with good prognosis.^2^, ^4^, ^6^, ^7^, ^8^, ^9^, ^10^ Although mostly performed in asymptomatic individuals, there is evidence that CAC scoring also has prognostic value in symptomatic populations.^11^, ^12^, ^13^, ^14^

Assessment of the extent of CAC is of importance for prediction as well as for diagnostic and therapeutic considerations.^15^ Beyond the Agatston calcium score, other characteristics, such as the calcium area, volume, mass, and density of plaques in coronary arteries, can be obtained from coronary calcium scanning.^16^ Several studies have shown associations between CAC density and risk of cardiovascular events^17^ and mortality.^17^, ^18^ However, little is known about the predictive value of specific characteristics of CAC in symptomatic individuals. The aim of this study is to determine to what extent these specific CAC characteristics are associated with major adverse cardiac events (MACEs) in symptomatic individuals suspected of having coronary artery disease (CAD).

## Methods

The data, analytic methods, and study materials will not be made available to other researchers for purposes of reproducing the results or replicating the procedure.

### Study Participants

Symptomatic individuals, aged 45 to 85 years, referred for clinically driven invasive coronary angiography (ICA) for suspected or known CAD, were enrolled at 16 sites in 8 countries in the prospective, multicenter, international CORE320 (Combined Noninvasive Coronary Angiography and Myocardial Perfusion Imaging Using 320 Detector Computed Tomography) study (http://www.clinicaltrials.gov, NCT00934037). This trial examined the diagnostic accuracy of combined 320‐row CT angiography (CTA) and myocardial CT perfusion imaging in comparison to the combination of ICA and single‐photon emission CT myocardial perfusion imaging. The study design and CT methods have been previously reported.^19^, ^20^ All participants underwent a CAC scan and cardiac CTA before clinically indicated ICA. Informed consent was given by all patients, and the study was approved by the institutional review boards of all participating sites.

### CT Acquisition

A detailed description of the CT acquisition and the interpretation methods has been previously published.^19^ In brief, a noncontrast cardiac CT scan was performed before the CTA on a 320×0.5‐mm detector row CT system (Aquilion ONE; Toshiba Medical Systems, Otawara, Japan). For CAC measurements, noncontrast cardiac CT scans were performed using prospective ECG triggering over a single heartbeat with a gantry rotation and x‐ray exposure time of 0.35 seconds and 0.5‐mm slice collimation. The CTA scan was performed using prospective triggering. All images were reconstructed in a centralized core laboratory and interpreted in a blinded core laboratory.

### Coronary calcium quantification

#### Coronary atherosclerotic calcium quantification

In the noncontrast scan, Agatston score and CAC volume were measured on a commercially available CT workstation (Vitrea, FX version 3.0 workstation; Vital Images, Minnetonka, MN). We computed scores for Agatston, volume, area, mass, and density (Figure).

1. The Agatston score was computed using standard methods.^1^
2. The volume score was computed as the sum of the total volume of calcium of the calcified regions in all vessels. The actual volume score measure, first introduced by Callister et al,^21^ is 1/1000th of a cubic centimeter; however, it is artificially multiplied by 1000 to make it more comparable to the Agatston score.
3. The area score was computed by dividing the calcium volume score by the reconstruction slice thickness of 3 mm.
4. The mass score was computed on the Vitrea workstation using standard methods with a calibration factor of 0.838.^16^
5. The mean density score was computed by dividing the Agatston score by the area score.^17^

**Figure 1:**
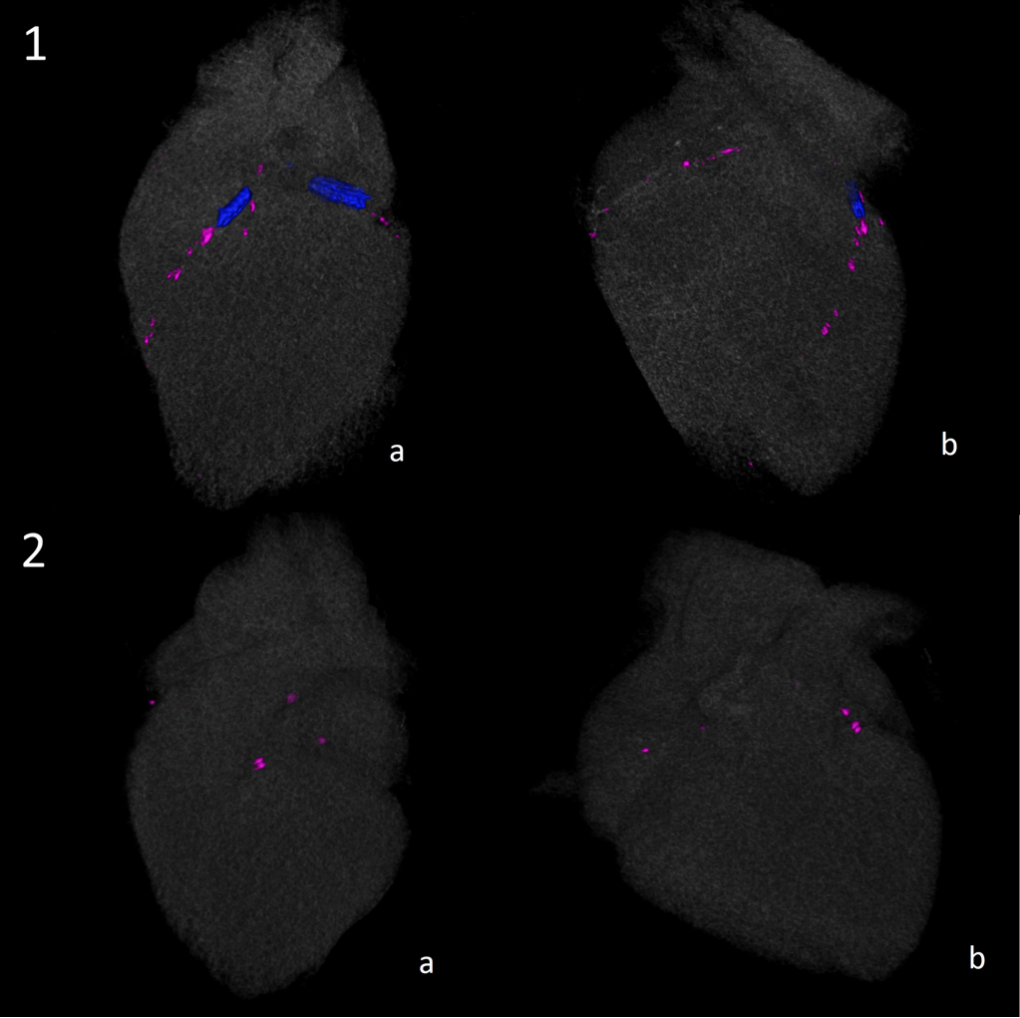
An illustration of 2 cases with comparable Agatston scores and different coronary artery calcium (CAC) density scores. Panel 1 depicts cardiac computed tomographic images, showing low‐density calcified plaque of a 44‐year‐old symptomatic man, with a body mass index of 26.5 kg/m^2^, referred for invasive angiography for suspected coronary artery disease. The Agatston score and CAC density score are 113 and 1.67, respectively. Panel 1 includes stented segments that were not calculated as part of the coronary calcium score. Panel 2 depicts similar images of a 64‐year‐old symptomatic woman, with a body mass index of 24.8 kg/m^2^, with high‐density calcified plaque, yielding an Agatston score of 107 and a CAC density score of 3.57. A, The left coronary circulation. B, The right coronary circulation.

### Major Adverse Cardiac Events

The primary outcome was time to MACEs at 2 years. MACE was defined as a composite of the occurrence of myocardial infarction, cardiac death, hospitalization for chest pain or congestive heart failure, arrhythmia, and late revascularization (beyond 182 days of the index ICA). Events were ascertained at 30 days, 6 months, 12 months, and 24 months after enrollment. Follow‐up data were acquired through office visits, telephone interviews, and medical record review using a standardized questionnaire. A panel of 9 physicians adjudicated the reported events. For adjudication, non‐English medical records were translated to English. Twenty‐one had 1 MACE, and 11 patients had 1 or 2 repeated events.

### Statistical Analysis

Spearman correlation coefficients were used to describe the associations between Agatston, volume, area, mass, and density scores. Agatston, volume, area, and mass scores were modelled as natural logarithms (log [score+1]) because of their right‐skewed distributions. The addition of 1 to the calcium score before logarithmic transformation allowed us to include all patients with a calcium score of 0 in the analyses. All predictors were then rescaled by dividing by their SD to facilitate interpretation of the results. Three proportional means models with a robust variance estimate were used to quantify the independent associations of CAC scores (Agatston, volume, area, mass, and density scores) and diameter stenosis with recurrent MACEs. The first model was unadjusted. Potential confounding effects of age, sex, and race were accounted for in the second model, and the third model additionally included baseline statin use. In addition, a sensitivity analysis excluding patients with a CAC score of 0 was performed, using the same proportional means models. The incremental value of density and diameter stenosis above Agatston score to predict time to MACEs was evaluated using nested proportional means models, adjusting for age, sex, and race. Again, a sensitivity analysis excluding patients with a negative CAC score was performed. Proportionality of hazards was tested in all models, and all *P* values were >0.30. All hazard ratios models reflected 1‐SD difference in the main exposures. Statistical analyses were performed using SAS, version 9.4 (SAS Institute, Cary, NC). All tests were 2 sided, and *P*<0.05 was considered statistically significant.

## Results

All 379 patients completed follow‐up, underwent CAC scanning and CTA, and were available for the analysis. The median age of the population was 62 years (interquartile range [IQR], 56–68 years), 34% were women, 56% were white, 33% were Asian, and 10% were black (Table 1). Agatston CAC score >0 was found in 314 patients. Median Agatston score was 162 units (IQR, 9–548 units), median volume score was 159 (IQR, 11–472), median area score was 53 (IQR, 4–157), median mass score was 34 (IQR, 2–109), and median density score was 3 (IQR, 2–4). A total of 46 cases of MACEs occurred in 32 of the 379 participants included (8.4%), of which 30 were revascularizations beyond 182 days of the index ICA, 5 were myocardial infarctions, 1 was a cardiac death, 1 was a hospitalization for congestive heart failure, 8 were hospitalizations for chest pain, and 1 was a case of arrhythmia. Eight patients experienced 2 MACEs, and 3 experienced a total of 3 MACEs over 2 years of follow‐up.

**Table 1:** Baseline Characteristics

Characteristics	All (N=379)	Agatston Calcium Score			
		0 (N=64)	1–100 (N=93)	101–399 (N=100)	≥400 (N=121)
Age, y	62.0 (55.6–68.4)	57.6 (51.6–61.8)	60.8 (54.4–67.8)	63.5 (58.3–68.4)	63.5 (57.9–71.2)
Male sex	252 (66.5)	27 (42.2)	49 (52.7)	75 (75.0)	100 (82.6)
Race					
White	216 (56.2)	37 (57.8)	52 (55.9)	54 (54.0)	69 (57.0)
Black	39 (10.3)	12 (18.8)	12 (12.9)	7 (7.0)	8 (6.6)
Asian	123 (32.5)	14 (21.9)	29 (31.2)	37 (37.0)	43 (35.5)
Other	4 (1.1)	1 (1.6)		2 (2.0)	1 (0.8)
Clinical characteristics					
Body mass index, kg/m^2^	26.6 (24.1–30.1)	26.6 (24.2–30.5)	27.1 (24.6–30.3)	26.2 (23.9–28.9)	26.6 (24.2–30.1)
Hypertension	295 (78.2)	34 (54.8)	74 (79.6)	84 (84.0)	102 (84.3)
Diabetes mellitus	130 (34.3)	16 (25.0)	32 (34.4)	35 (35.0)	46 (38.0)
Dyslipidemia	252 (67.9)	32 (50.0)	58 (64.4)	68 (69.4)	93 (78.8)
Previous myocardial infarction	103 (27.2)	8 (12.5)	30 (32.3)	28 (28.0)	37 (30.6)
Statin use	204 (68.7)	33 (67.4)	42 (59.2)	54 (68.4)	74 (77.3)
Smoking					
Current	62 (17.1)	11 (17.2)	13 (14.8)	21 (22.6)	17 (14.7)
Former	133 (36.7)	19 (29.7)	32 (36.4)	35 (37.6)	46 (39.7)
Never	167 (46.1)	34 (53.1)	43 (48.9)	37 (39.8)	53 (45.7)
Family history of CAD	161 (45.2)	25 (39.1)	32 (37.2)	45 (46.4)	58 (53.7)
Prior PCI	113 (29.8)	7 (10.9)	29 (31.2)	32 (32.0)	45 (37.2)
History of unstable angina	27 (7.3)	3 (4.8)	4 (4.4)	6 (6.0)	14 (12.0)
Angina (30 d), Canadian class					
0	62 (21.5)	19 (34.5)	17 (25.4)	14 (17.3)	12 (14.3)
1	110 (38.2)	23 (41.8)	28 (41.8)	28 (34.6)	31 (36.9)
2	98 (34.0)	13 (23.6)	17 (25.4)	32 (39.5)	35 (41.7)
3	14 (4.9)		4 (6.0)	6 (7.4)	4 (4.8)
4	4 (1.4)		1 (1.5)	1 (1.2)	2 (2.4)
Scores					
Agatston	162 (9–548)	0 (0–0)	18 (5–54)	214 (154–295)	865 (559–1420)
Volume	159 (11–472)	0 (0–0)	23 (9–58)	203 (145–274)	767 (484–1153)
Area	53 (4–157)	0 (0–0)	8 (3–19)	68 (48–91)	256 (161–384)
Mass	34 (2–109)	0 (0–0)	4 (1–11)	45 (29–61)	178 (114–301)
Density	3 (2–4)	0 (0–0)	2 (2–3)	3 (3–4)	4 (3–4)
Stenosis, %	62 (41–89)	36 (15–42)	43 (35–58)	71 (52–93)	87 (67–100)
MACEs per 1000 patient‐years	44.9 (31.7–63.4)	16.2 (4.0–64.7)	33.7 (15.1–74.9)	58.6 (32.5–105.9)	58.7 (34.1–101.0)

Values are number (percentage) or median (interquartile range). CAD indicates coronary artery disease; MACE, major adverse cardiac event; PCI, percutaneous coronary intervention.

### CAC Scores and Diameter Stenosis as Predictors of MACEs

All CAC scores (Agatston, volume, area, mass, and density) were strongly correlated, with *r*>0.80 for correlations between calcium density and the other scores and *r*>0.99 for correlations between Agatston, volume, area, and mass scores (Table 2). In unadjusted models, Agatston score, CAC volume, CAC area, CAC mass, density score, and diameter stenosis were all significant independent predictors of MACEs, with diameter stenosis being the strongest predictor (Table 3). In models adjusted for age, sex, and race, the Agatston score, density score, and diameter stenosis were significant predictors of MACEs, with CAC density being the strongest predictor among the CAC scores, with a hazard ratio of 1.70 (95% confidence interval [CI], 1.09–2.65) compared with Agatston score, with a hazard ratio of 1.45 (95% CI, 0.99–2.10) (model 1, Table 3). After a further adjustment for statin use (model 2), CAC density remained the strongest predictor of MACEs among CAC scores. The strongest overall predictor of MACEs in the adjusted models (models 1 and 2) was diameter stenosis. In the sensitivity analysis excluding patients with a CAC score of 0, the associations between CAC scores (Agatston, volume, area, mass, and density) and MACEs remained positive, but were no longer significant (Table 4).

**Table 2:** Spearman Correlation for the 5 CAC Scores

Score	ln (Agatston)	ln (Volume)	ln (Area)	Density	ln (Mass)
ln (Agatston)	1.00	0.997	0.998	0.832	0.998
ln (Volume)		1.00	1.00	0.800	0.996
ln (Area)			1.00	0.800	0.996
Density				1.00	0.835
ln (Mass)					1.00

CAC indicates coronary artery calcium; ln, natural logarithm.

**Table 3:** CAC Scores (Agatston, Volume, Area, Mass, and Density) and Diameter Stenosis as Predictors of MACEs

Scores	Median (IQR)		Unadjusted Model		Model 1		Model 2	
	No Event (N=347)	Event (N=46)	HR (95% CI)	*P* Value	HR (95% CI)	*P* Value	HR (95% CI)	*P* Value
ln (Agatston), per SD	2.0 (0.7–2.4)	2.3 (1.8–2.5)	1.49 (1.07–2.07)	0.02	1.45 (0.99–2.10)	0.05	1.45 (1.00–2.10)	0.05
ln (Volume), per SD	2.0 (0.9–2.5)	2.3 (1.8–2.6)	1.50 (1.06–2.12)	0.02	1.44 (0.99–2.10)	0.06	1.46 (1.01–2.10)	0.05
ln (Area), per SD	1.9 (0.7–2.4)	2.2 (1.6–2.5)	1.44 (1.04–2.02)	0.03	1.38 (0.95–1.99)	0.09	1.39 (0.98–1.99)	0.07
ln (Mass), per SD	1.7 (0.4–2.3)	2.0 (1.4–2.3)	1.37 (1.00–1.88)	0.05	1.32 (0.93–1.87)	0.13	1.34 (0.95–1.88)	0.10
Density, per SD	2.3 (1.3–2.7)	2.6 (2.2–2.7)	1.66 (1.11–2.49)	0.01	1.70 (1.09–2.65)	0.02	1.63 (1.04–2.58)	0.03
Stenosis, per SD, %	2.2 (1.4–3.1)	3.1 (2.0–3.6)	1.93 (1.36–2.74)	0.0002	2.06 (1.23–3.43)	0.006	2.12 (1.26–3.57)	0.005

Model 1, adjusted for age, sex, and race; model 2, model 1+adjusted for statin use. CAC indicates coronary artery calcium; CI, confidence interval; HR, hazard ratio; IQR, interquartile range; ln, natural logarithm; MACE, major adverse cardiac event.

**Table 4:** CAC Scores (Agatston, Volume, Area, Mass, and Density) and Diameter Stenosis as Predictors of MACEs in Patients With CAC >0

Scores	Median (IQR)		Unadjusted Model		Model 1		Model 2	
	No Event (N=285)	Event (N=30)	HR (95% CI)	*P* Value	HR (95% CI)	*P* Value	HR (95% CI)	*P* Value
ln (Agatston), per SD	2.1 (1.6–2.5)	2.3 (1.8–2.5)	1.24 (0.79–1.95)	0.35	1.15 (0.70–1.90)	0.58	1.17 (0.71–1.94)	0.54
ln (Volume), per SD	2.2 (1.7–2.6)	2.3 (2.0–2.6)	1.24 (0.77–1.98)	0.37	1.12 (0.65–1.91)	0.68	1.16 (0.68–1.96)	0.59
ln (Area), per SD	2.1 (1.5–2.5)	2.2 (1.8–2.5)	1.19 (0.79–1.81)	0.41	1.09 (0.67–1.75)	0.73	1.12 (0.70–1.79)	0.64
ln (Mass), per SD	1.9 (1.2–2.4)	2.0 (1.5–2.4)	1.14 (0.78–1.67)	0.49	1.06 (0.69–1.63)	0.79	1.09 (0.71–1.66)	0.70
Density, per SD	2.4 (2.0–2.7)	2.6 (2.2–2.7)	1.45 (0.80–2.66)	0.22	1.48 (0.70–3.11)	0.30	1.40 (0.64–3.10)	0.40
Stenosis, per SD, %	2.4 (1.7–3.3)	3.1 (2.0–3.6)	1.78 (1.21–2.62)	0.004	1.89 (1.07–3.35)	0.03	1.98 (1.11–3.54)	0.02

Model 1, adjusted for age, sex, and race; model 2, model 1+adjusted for statin use. CAC indicates coronary artery calcium; CI, confidence interval; HR, hazard ratio; IQR, interquartile range; ln, natural logarithm; MACE, major adverse cardiac event.

### Incremental Value of CAC Density and Diameter Stenosis Above Agatston Score in Predication of MACEs

Agatston score was a significant predictor of MACEs in symptomatic patients after adjustment for age, sex, and race, with a hazard ratio of 1.45 (95% CI, 0.99–2.10); however, this association was no longer significant when accounting for CAC density in the model (Table 5). A further adjustment for diameter stenosis yielded an estimated hazard ratio of 0.41 (95% CI, 0.19–0.87), suggesting a protective effect of Agatston score on MACEs after adjustment, whereas calcium density and diameter stenosis remained positively associated with the risk of MACEs (adjusted hazard ratios, 2.62 [95% CI, 1.00–6.82] and 1.03 [95% CI, 1.01–1.05], respectively). After excluding patients with a CAC score of 0, similar estimates were found, except for the association between Agatston score and MACEs, when adjusted for age, sex, and race (hazard ratio, 1.15 [95% CI, 0.70–1.90]).

**Table 5:** Incremental Value of CAC Density and Diameter Stenosis Above Agatston Score to Predict 2‐Year MACEs in Symptomatic Patients

Variable	Model 1		Model 2		Model 3	
	HR (95% CI)	*P* Value	HR (95% CI)	*P* Value	HR (95% CI)	*P* Value
ln (Agatston), per 1 SD	1.45 (0.99–2.10)	0.05	0.82 (0.35–1.95)	0.66	0.41 (0.19–0.87)	0.02
Density, per 1 SD			2.02 (0.80–5.09)	0.14	2.62 (1.00–6.82)	0.05
Stenosis, per 1 SD, %					1.03 (1.01–1.05)	0.002

All models are adjusted for age, sex, and race. CAC indicates coronary artery calcium; CI, confidence interval; HR, hazard ratio; ln, natural logarithm; MACE, major adverse cardiac event.

## Discussion

In our international study population with symptomatic individuals, different characteristics of CAC were measured to investigate their associations with incident MACEs. Our main findings are as follows: (1) Agatston score, calcium volume score, and calcium density score are significantly and independently associated with risk of 2‐year MACEs in a population with suspected CAD; (2) in our symptomatic population, CAC density is a stronger predictor than Agatston CAC scoring for risk prediction of incident MACEs; and (3) CAC density does not provide incremental predictive ability beyond the Agatston score and diameter stenosis in predicting 2‐year MACEs.

This is, to our knowledge, the first study on the independent associations of Agatston CAC score together with calcium volume, area, mass, and density scores of coronary artery plaques, with incident MACEs in symptomatic patients. More insight into these associations can be of value when evaluating current CAC scoring systems and assessing the role of CAC scores in coronary heart disease risk prediction models for symptomatic patients. Although the use of CTA for prognosis of CAD keeps evolving,^22^ risk prediction through CAC scanning similarly is subject to promising developments. In a recent study, Blaha et al found that the number of coronary arteries with calcified plaques adds significantly to the traditional Agatston CAC score for prediction of cardiac events.^23^ With our study, we aimed to improve the ability of traditional Agatston calcium scoring to predict cardiac events by addressing the different characteristics of the CAC score.

Coronary calcium scanning is a well‐known risk prediction method that is widely used in asymptomatic patients. We report that, in our symptomatic population, CAC density is superior to traditional Agatston CAC scoring for risk prediction of MACEs. Similarly, several studies have demonstrated that CAC scoring provides predictive value in symptomatic populations.^11^, ^12^, ^13^ In 2012, Hou et al^13^ examined the prognostic value of cardiac CTA and calcium score for MACEs in a cohort that was mainly composed of symptomatic outpatients. They concluded that CAC scoring is not only of predictive value, but also has incremental value over routine risk factors for MACEs. More recently, Nicoll et al^24^ studied >5000 symptomatic patients and found that the Agatston score is a more accurate predictor of significant (>50%) coronary stenosis than conventional risk factors.

Of the different CAC scores, CAC density was independently the most predictive in our analyses. Consistent with our data, a recently published study in patients receiving hemodialysis by Bellasi et al reported plaque density as an independent predictor of all‐cause mortality.^18^

However, when comparing this finding with an asymptomatic population, the MESA (Multi‐Ethnic Study of Atherosclerosis) showed an inverse association between the calcium density of atherosclerotic plaques and risk of cardiovascular events,^17^, ^25^ indicating a protective effect of plaques with greater calcium density. Similarly, prior studies have shown higher CAC density in patients with stable cardiovascular disease, compared with plaques in patients with acute heart disease,^26^, ^27^ suggesting that increased plaque density might be a sign of stabilization of disease. However, there are some important differences that may explain the difference in association of CAC density with MACEs. In the asymptomatic population studies, the definition of MACEs included only hard outcomes (death, myocardial infarction, and stroke), whereas in our study, we included revascularization in the definition of MACEs. All revascularizations, however, were from chronic symptoms, because acute coronary syndrome was an exclusion criterion in the CORE320 study. This was another important difference for the patient populations. It is plausible that less dense plaques are more likely to rupture in otherwise asymptomatic patients, whereas the denser plaques are more likely to be older and associated with stable CAD symptoms, therefore more often leading to revascularizations. This could mean that the CAC density could be an indication of the disease stage in the symptomatic patients. Last, the MESA investigators reported the association between CAC density and cardiac events after adjusting CAC density for CAC volume score, whereas in our study, CAC density was adjusted for traditional risk factors and statin use. This could mean that the low‐density score mainly adds predictive value in patients with smaller plaques and, thus, that the extent of disease, reflected in the CAC volume score, is leading. However, we strongly believe further research is necessary to test these hypotheses.

Multiple studies have shown that absence of CAC is associated with good prognosis in low‐risk populations.^2^, ^4^, ^6^, ^7^, ^8^, ^9^ In high‐risk populations, however, a 0 calcium score may be seen in almost 20% of patients with obstructive CAD in need of revascularization.^28^ More recently, analyses within symptomatic patients from CONFIRM (Coronary CT Angiography Evaluation for Clinical Outcomes: An International Multicenter Registry) showed that CAC absence does significantly reduce, but does not fully eliminate, the occurrence of obstructive CAD.^29^

Our study is not without limitations. The CORE320 study was designed as a diagnostic accuracy study and may not be powered for this analysis and hard events in particular. Most of our events were revascularization rather than hard events (death, myocardial infarction, and stroke). Another potential limitation is that the mean CAC density score was relatively high with limited variability, leading to a high correlation between the CAC density and the Agatston score. This could have made it more difficult to differentiate between the scores. The association of Agatston score in this model is clearly highly influenced by the strong positive associations among these variables, because the association in a multivariable model is really the residual effect of each predictor after adjustment for the other predictors in the model. In particular, if each of the Agatston and density scores is adjusted for the other, the remaining “unique contribution” residual variables are highly negatively correlated. Given these relationships, the protective effect of the Agatston score in the multivariable model is highly questionable, because it is not really an independent effect.

The results of this study support the potential of CAC scanning for risk stratification in symptomatic individuals. Nevertheless, we believe cautiousness about the implications of our conclusion is appropriate and further study in a practical clinical trial is necessary to confirm the application of CAC scanning for this purpose. Although greater CAC density appears to be protective in asymptomatic patients without known CAD*,* greater CAC density might predict need for revascularization in symptomatic patients. More research into CAC scoring and the predictive value of different CAC characteristics in symptomatic patients is essential. Comparison between the Agatston score and CAC density score as well as the influence of CAC scores when added to existing risk scores for patients suspected of having CAD are areas where further exploration could well be meaningful.

## Conclusion

Agatston score, calcium volume score, and calcium density score are significant and independent predictors of incident 2‐year MACEs in symptomatic individuals, with calcium density being superior to Agatston and calcium volume scoring.

## Sources of Funding

The study was funded by Toshiba Medical Systems.

## Disclosures

Arbab‐Zadeh is a Steering Committee member of the CORE320 (Combined Noninvasive Coronary Angiography and Myocardial Perfusion Imaging Using 320 Detector Computed Tomography) study. Schuijf is an employee of Toshiba Medical Systems. Miller receives honoraria from Toshiba Medical Systems and institutional grants from Toshiba Medical Systems and Doris Duke Charitable Foundation; the institution receives support for travel to study meetings and educational purposes from Toshiba Medical Systems. Lima is Principal Investigator of Grants from Toshiba Medical Systems and Bracco Diagnostics, which, in part, support the CORE320 study. The remaining authors have no disclosures to report.
